# Synthesis and Antifungal Activity of Metal Complexes Containing Dichloro-Tetramorpholino-Cyclophosphazatriene

**DOI:** 10.1155/MBD.1998.287

**Published:** 1998

**Authors:** Cornelia Guran, Mihai Barboiu, Paula Diaconescu, Vlad Iluc, Mihaela Bojin, Andrea Scozzafava, Claudiu T. Supuran

**Affiliations:** 1 lnorganic Chemistry Department Polytechnic University of Bucharest 1 Polizu Street Bucharest R-78126 Romania; 2 Research Centre for Macromolecular Materials and Membranes Splaiul Independentei 206 Bucharest R-79611 Romania; 3 Università degli Studi Dipartimento di Chimica, Laboratorio di Chimica Inorganica e Bioinorganica Via Gino Capponi 7 Florence I-50121 Italy

## Abstract

Metal complexes of dichloro-tetramorpholino-cyclophosphazatriene containing divalent cations such as Ni(II), Co(II), and Mn(II) have been prepared and characterised by standard physico-chemical procedures (elemental chemical analysis, IR and UV-VIS spectra, conductimetric measurement). The newly synthesised compounds possessed antifungal activity against *Aspergillus* and *Candida spp*., some of them showing effects comparable to ketoconazole (with minimum inhibitory concentrations in the range of 2- 30 μg/mL) but being generally less active as compared to the azole. Best activity was detected against *C*. *albicans*, and worst activity against *A*. *niger*. The mechanism of action of these compounds probably involves inhibition of ergosterol biosynthesis, and interaction with lanosterol-14-α-demethylase (CYP51A1), since reduced amounts of ergosterol were evidenced by means of HPLC in cultures of the sensitive strain *A*. *niger* treated with some of these inhibitors.


### SYNTHESIS AND ANTIFUNGAL ACTIVITY OF METAL COMPLEXES CONTAINING DICHLORO-TETRAMORPHOLINO-CYCLOPHOSPHAZATRIENE

Cornelia Guran[1], Mihai Barboiu[2], Paula Diaconescu[1], Vlad Iluc[1], Mihaela Bojin[1], Andrea Scozzafava[3] and Claudiu T. Supuran*[3]

[1]Inorganic Chemistry Department, Polytechnic University of Bucharest, 1 Polizu Street, R-78126 Bucharest, Roumania
[2]Research Centre for Macromolecular Materials and Membranes, Splaiul Independentei 206, R-79611 Bucharest, Roumania
[3]Università degli Studi, Dipartimento di Chimica, Laboratorio di Chimica Inorganica e Bioinorganica, Via Gino Capponi 7, I-50121, Florence, Italy

**Abstract:** Metal complexes of dichloro-tetramorpholino-cyclophosphazatriene containing divalent cations such as Ni(II), Co(II), and Mn(II) have been prepared and characterised by standard physico-chemical procedures (elemental chemical analysis, IR and UV-VIS spectra, conductimetric measurement). The newly synthesised compounds possessed antifungal activity against *Aspergillus* and *Candida spp.*, some of them showing effects comparable to ketoconazole (with minimum inhibitory concentrations in the range of 2- 30 μg/mL) but being generally less active as compared to the azole. Best activity was detected against *C. albicans*, and worst activity against *A. niger*. The mechanism of action of these compounds probably involves inhibition of ergosterol biosynthesis, and interaction with lanosterol-14-α-demethylase (CYP51A1), since reduced amounts of ergosterol were evidenced by means of HPLC in cultures of the sensitive strain *A. niger* treated with some of these inhibitors.

### Introduction

Recent developments in the chemistry of phosphazenes were focused on the synthesis of novel types of linear, cyclic or polymeric such derivatives [1-4] and on their application as precursors for obtaining materials with special properties [5-8].

The study of the co-ordination chemistry of cyclophosphazene derivatives is of considerable interest because of the high versatility of these compounds as ligands, and the putative applications as catalysts of the obtained metal complexes [9]. Although the unsubstituted cyclophosphazene behaves as a poor ligand towards transition cations, the presence of moieties containing different heteroatoms attached on the cyclophosphazene ring highly increases their affinity for metal ions[10-14]. Few metal complexes containing cyclophosphazene derivatives as ligands have been reported up to now. Recently [15,16] this group reported the synthesis of 1,3,5-tris(8-hydroxyquinolino)-trichlorocyclophosphazatriene and of some of its complexes of the general formula $[MLCl_2]$, $[ML_2]Cl_2$, (M = Cu(II), Co(II), Ni(II)); [NiLAc], $[NiL_2Ac]Ac$, $[ML_3]X_3$ (M = Ni(II), Co(II), X = Cl, AcO). By using dichloro-tetramorpholino-cyclophosphazatriene (L) as ligand, a series of Cu(II) new complexes were also synthesised[16]. Some of these complexes[15] inhibited the growth of several fungi species, such as *Aspergillus* and *Candida spp.*

In this paper we extend our previous research[15] in the synthesis and antifungal evaluation of some metal complexes containing cyclophosphazene derivatives as ligands. Here we present the synthesis and characterisation of the $Ni^{2+}$, $Co^{2+}$ and $Mn^{2+}$ complexes of dichloro-tetramorpholino-cyclophosphazatriene (L) as well as biological activity studies with these new derivatives. The obtained complexes were assayed as inhibitors against several widespread fungi, such as *Aspergillus* and *Candida spp.*, evidencing interesting activity for some of them. For the most active compound against *C. albicans*, the amount of ergosterol after treatment with different concentrations of the new and azole type inhibitors have been determined by means of HPLC, being thus shown that the antifungal effect of the new class of compounds is probably indeed due to inhibition of ergosterol biosynthesis. It is thus probable that these derivatives and the azole antifungal compounds, such as ketoconazole or itraconazole possess a similar mechanism of action at molecular level.[17,18]

### Materials and Methods

Elemental analyses were done by combustion with a Carlo Erba Instrument or gravimetrically for the metal ions. Electronic spectra have been recorded at room temperature on a VSU-2G spectrophotometer using MgO as standard material. IR spectra were recorded with a Perkin-Elmer spectrophotometer using KBr pellets as reference. Conductimetric measurements were done at room temperature on a Radelkis KFT conductivity-meter. Dichloro-tetramorpholino-cyclophosphazatriene (L) was prepared as described in the literature[17].

*Synthesis of complexes of dichloro-tetramorpholino-cyclophosphazatriene (L)* ***1-7***

All complexes have been synthesised according to the following general procedure: methanolic solutions containing the metal salts $MX_2{\cdot}nH_2O$ (M = Co, Ni, Mn; X = Cl, AcO) and the ligand (L) were mixed with stirring in 1:1, 2:1 and 3:2 molar ratios of M : L. The resulting mixtures were refluxed for 60 min on a water bath. The complexes were separated by filtration, washed with diethylether and dried in a dessicator (on $(P_2O_5)_2$). The yields were around 60-70%. In each case the starting amount of L used in the synthesis was 0.001 moles (0.550g).

**[NiL(AcO)$_2$] 1** : IR(KBr): $\nu Ni_{-N}$ = 521cm$^{-1}$, $\nu_{P\text{-}Cl}$ = 600cm$^{-1}$, $\nu_{P\text{-}N}$ = 1209cm$^{-1}$, $\nu_{C\text{-}N\ aliphatic\ amine}$ = 1120, 1140cm$^{-1}$, $\rho_{CH2}$ = 847cm$^{-1}$, $\nu_{as\ CH3COO}$ = 1598cm$^{-1}$, $\delta_{HOH}$ = 1656, 3380cm$^{-1}$ (lattice water molecules). UV-VIS (CsBr): $\nu_1$ = 8.90kK ($^3A_{2g} \rightarrow {^3T_{2g}}(F)$), $\nu_2$ = 11.90kK ($^3A_{2g} \rightarrow {^3E_g}(^3T_{1g})$) and 15.20kK ($^3A_{2g} \rightarrow {^3B_{2g}}(^3T_{1g})$), $\nu_3$ = 18.20kK ($^3A_{2g} \rightarrow {^3T_{1g}}(P)$).M = 726.7, Calcd.: Ni 8.08, N 7.71, Cl 9.77 %; found Ni 7.86, N 7.45, Cl 10.03 %.

**[Ni$_2$L(AcO)$_4$] 2** : IR(KBr): $\nu_{Ni\text{-}N}$ = 521cm$^{-1}$, $\nu_{P\text{-}Cl}$ = 605cm$^{-1}$, $\nu_{P\text{-}N}$ = 1209cm$^{-1}$, $\nu_{C\text{-}N\ aliphatic\ amine}$ = 1115, 1140cm$^{-1}$, $\rho_{CH2}$ = 852cm$^{-1}$, $\nu_{as\ CH3COO}$ = 1588cm$^{-1}$, $\delta_{HOH}$ = 1660, 3385cm$^{-1}$ (lattice water molecules).UV-VIS (CsBr): $\nu_1$ = 9.09kK ($^3A_{2g} \rightarrow {^3T_{2g}}(F)$), $\nu_2$ = 12.14kK ($^3A_{2g} \rightarrow {^3E_g}(^3T_{1g})$) and 14.63kK ($^3A_{2g} \rightarrow {^3B_{2g}}(^3T_{1g})$), $\nu_3$ = 17.95kK ($^3A_{2g} \rightarrow {^3T_{1g}}(P)$).M = 903.4. Calcd.: Ni 12.99, N 6.20, Cl 7.86 %; found Ni 12.65, N 6.37, Cl 8.18 %.

**[Ni$_3$L$_2$(H$_2$O)$_2$(AcO)$_4$](AcO)$_2$ 3**: IR(KBr): $\nu_{Ni\text{-}N}$ = 521cm$^{-1}$, $\nu_{P\text{-}Cl}$ = 610cm$^{-1}$, $\nu_{P\text{-}N}$ = 1204cm$^{-1}$, $\nu_{C\text{-}N\ aliphatic\ amine}$ = 1115, 1140cm$^{-1}$, $\rho_{CH2}$ = 841cm$^{-1}$, $\nu_{as\ CH3COO}$ = 1582cm$^{-1}$, $\delta_{HOH}$ = 1660, 3390cm$^{-1}$ (coordination and lattice water molecules).UV-VIS (CsBr): $\nu_1$ = 8.21kK ($^3A_{2g} \rightarrow {^3T_{2g}}(F)$), $\nu_2$ = 11.48kK ($^3A_{2g} \rightarrow {^3E_g}(^3T_{1g})$) and 15.68kK ($^3A_{2g} \rightarrow {^3B_{2g}}(^3T_{1g})$), $\nu_3$ = 18.15kK ($^3A_{2g} \rightarrow {^3T_{1g}}(P)$), $\nu$ = 24.96kK (charge transfer).M = 1666.1. Calcd.: Ni 10.57, N 6.72, Cl 8.52 %; found: Ni 10.22, N 6.20, Cl 9.04 %.

**[CoLCl$_2$] 4**: IR(KBr): $\nu_{Co\text{-}N}$ = 510cm$^{-1}$, $\nu_{Co\text{-}Cl}$ = 490cm$^{-1}$, $\nu_{P\text{-}Cl}$ = 600cm$^{-1}$, $\nu_{P\text{-}N}$ = 1190cm$^{-1}$, $\nu_{C\text{-}N\ aliphatic\ amine}$ = 1120, 1136cm$^{-1}$, $\rho_{CH2}$ = 847cm$^{-1}$, $\delta_{HOH}$ = 1640, 3417cm$^{-1}$ (lattice water molecules).UV-VIS (CsBr): $\nu_2$ = 6.15kK ($^4A_2 \rightarrow {^4T_1}(F)$), $\nu_3$ = 15.35kK ($^4A_2 \rightarrow {^4T_1}(P)$).M = 679.9. Calcd. Co = 8.66, N = 8.24, Cl = 20.88 %; found: Co 8.15, N= 7.95, Cl 21.14 %.

**[Co$_2$LCl$_4$] 5**: IR(KBr): $\nu_{Co\text{-}N}$ = 510cm$^{-1}$, $\nu_{Co\text{-}Cl}$ = 490cm$^{-1}$, $\nu_{P\text{-}Cl}$ = 605cm$^{-1}$, $\nu_{P\text{-}N}$ = 1194cm$^{-1}$, $\nu_{C\text{-}N\ aliphatic\ amine}$ = 1125, 1139cm$^{-1}$, $\rho_{CH2}$ = 851cm$^{-1}$, $\delta_{HOH}$ = 1634, 3405cm$^{-1}$ (lattice water molecules).UV-VIS (CsBr): $\nu_2$ = 6.75kK ($^4A_2 \rightarrow {^4T_1}(F)$), $\nu_3$ = 15.55kK ($^4A_2 \rightarrow {^4T_1}(P)$). $M_{th}$ = 809.8. Calcd. Co = 14.55, N = 6.91, Cl = 26.30 %; found: Co = 14.02; N 6.33, Cl 25.72 %.

**[Co$_3$L$_2$Cl$_4$]Cl$_2$ 6**: IR(KBr): $\nu_{Co\text{-}N}$ = 510cm$^{-1}$, $\nu_{Co\text{-}Cl}$ = 490cm$^{-1}$, $\nu_{P\text{-}Cl}$ = 610cm$^{-1}$, $\nu_{P\text{-}N}$ = 1198cm$^{-1}$, $\nu_{C\text{-}N\ aliphatic\ amine}$ = 1125, 1142cm$^{-1}$, $\rho_{CH2}$ = 852cm$^{-1}$, $\delta_{HOH}$ = 1640, 3412cm$^{-1}$ (lattice water molecules).UV-VIS (CsBr): $\nu_2$ = 6.70kK ($^4A_2 \rightarrow {^4T_1}(F)$), $\nu_3$ = 15.40kK ($^4A_2 \rightarrow {^4T_1}(P)$). M = 1489.7. Calcd. Co 11.86, N 7.52, Cl 19.06 %; found: Co 11.27, N 7.18, Cl 19.82 %.

**[MnL(OH$_2$)Cl$_2$] 7**: IR(KBr): $\nu_{Mn\text{-}N}$ = 515cm$^{-1}$, $\nu_{Mn\text{-}Cl}$ = 485cm$^{-1}$, $\nu_{P\text{-}Cl}$ = 600cm$^{-1}$, $\nu_{P\text{-}N}$ = 1202cm$^{-1}$, $\nu_{C\text{-}N\ aliphatic\ amine}$ = 1115, 1141cm$^{-1}$, $\rho_{CH2}$ = 847cm$^{-1}$, $\delta_{HOH}$ = 1652, 3387cm$^{-1}$ (coordination and lattice water molecules).UV-VIS (CsBr): $\nu_1$ = 14.89kK ($^6A_1 \rightarrow {^4T_{1g}}$), $\nu_2$ = 17.24kK ($^6A_1 \rightarrow {^4T_{2g}}$), $\nu_3$ = 19.44kK ($^6A_1 \rightarrow {^4A_{1g}}, {^4E_g}$). M = 711.9. Calcd. Mn 7.71, N 7.87, Cl = 19.95%; found Mn 8.36, N = 7.28, Cl 21.14 %.

*Assay of fungistatic activity of compounds* **1-7**

Fungistatic activity was determined by a modification of the growth method recently reported by us,[16,20-23] utilising two *Aspergillus* and one *Candida spp.* Minimum inhibitory concentrations (MICs) have been determined by the agar dilution method with Iso-Sensitest agar as described by Kinsman et al.[23] The fungi/moulds were cultivated in agar plates at 37°C for 5 days, in the nutrient broth (NB, Diagnostic Pasteur), in the absence and in the presence of 100 - 0.01 µg/mL of compounds **1-7**. Stock solutions of inhibitors were obtained in DMSO (100 mg/mL) and dilutions up to 0.01 µg/mL were done with distilled deionized water. The minimum concentration at which no growth was observed was taken as MIC value (µg/mL), and represents the mean of at least two determinations.

*Assay of sterols present in the fungi cultures*

A reverse-phase HPLC method adapted from the literature,[22] has been used to determine the amount of sterols (ergosterol and lanosterol) present in the fungi cultures. The fungi have been cultivated as mentioned above for 5 days, with or without inhibitors added in the nutrient broth. Culture media were suspended in a small volume of MOPS buffer (pH 7.4) and the cells centrifuged at 20000g for 30 min. Cells were weighed (wet paste) and broken by sonication. Sterols present in the homogenate were then extracted in chloroform, the solvent has been evaporated to a small volume and the extracts applied on a μ-Bondapak-C18 column, with acetonitrile as eluting solvent. Authentic ergosterol and lanosterol (from Sigma) were used as standards. The flow rate was of 3 mL/min. The retention times were: 8.87 min for ergosterol; and 7.62 min for lanosterol, respectively. Blank assay were done for cultures which were not treated with inhibitors in order to assess the normal levels of sterols present. The amount of ergosterol present in the same amount of wet cells from the culture grown in the absence of inhibitor was taken as 100%.[21-24]

**Results and Discussion**

The ligand L probably acts polydentately in its metal complexes, due to the presence of several exocyclic nitrogen, oxygen and chlorine as well as three endocyclic nitrogen atoms in its molecule. To establish the electronic density of different donor atoms present in the ligand, the dichloro-tetramorpholino-cyclophosphazatriene has been investigated with the Hyperchem programme.[25] As seen from Fig.1 the morpholine nitrogen and oxygen atoms have partial charges of -0.899 and -0.265, respectively, the chlorine atoms of -0.636, whereas the endocyclic nitrogens of the phosphazene ring, although the most negatively-charged (with a partial electronic density in the range -1.882/ -1.926), are probably less available for interaction with the metal ions due to the strong steric hindrance.

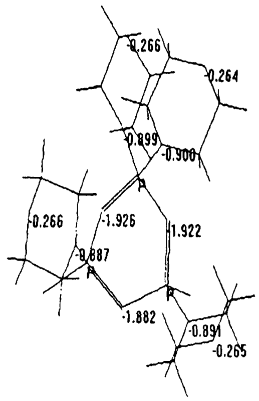

Fig. 1. Charge densities of the ligand L as calculated by means of the programme Hyperchem.

From data of Fig. 1, it can be estimated that in the mononuclear complexes **1**, **4** and **7** the ligand L probably acts bidentately by means of two morpholine nitrogen atoms bound to two different phosphorus atoms, while in the di- and trinuclear complexes **2**, **3**, **5**, and **6**, all the exocyclic nitrogen atoms of the ligand are involved in co-ordination of the metal ions

As seen from data of Table 1, the prepared complexes are non-electrolytes (**1**, **2**, **4**, **5**, and **7**), or 1:2 electrolytes (complexes **3** and **6**).

Table 1. Conductimetric data for the prepared complexes **1-7**.

| No | Complex | Molar conductance ($\Omega$ cm$^2$ mol$^{-1}$) | Electrolyte type | Colour |
|---|---|---|---|---|
| **1** | $[NiL(AcO)_2]$ | 52 | non-electrolyte | Green |
| **2** | $[Ni_2L(AcO)_4]$ | 48 | non-electrolyte | Green |
| **3** | $[Ni_3L_2(OH_2)_2(AcO)_4](AcO)_2$ | 145 | 1 : 2 | Green |
| **4** | $[CoLCl_2]$ | 35 | non-electrolyte | Blue |
| **5** | $[Co_2LCl_4]$ | 54 | non-electrolyte | Blue |
| **6** | $[Co_3L_2Cl_4]Cl_2$ | 128 | 1 : 2 | Blue |
| **7** | $[MnL(OH_2)_2Cl_2]$ | 27 | non-electrolyte | Light green |

The prepared complexes were also characterised by electronic and IR spectroscopy (Table 2).

For the complexes $[NiL(AcO)_2]$ (**1**), $[Ni_2L(AcO)_4]$ (**2**) and $[Ni_3L_2(OH_2)_2(AcO)_4](AcO)_2$ (**3**), the electronic spectra indicate the presence of octahedral Ni(II) ions. The $\nu_1$ band appears in the range of 8.2 kK whereas the $\nu_2$ as a large, splitted band, in the region 12.00-16.00 kK, characteristic for Ni(II) in octahedral surrounding.[26] The $\nu_3$ band associated to the $^3A_{2g}\rightarrow^3T_{1g}(P)$ transition, is probably superposed with a charge transfer band. The $\nu_2$ transition is resolved in two bands, an intense one at 11.48kK, assigned to the

$^3A_{2g}{\rightarrow}^3E_g(T_{1g})$ transition, and a second one of lower intensity, at 15.68 kK, assigned to the $^3A_{2g}{\rightarrow}^3B_{2g}(T_{1g})$ transition. The $\nu_1$ band has been assigned to the $^3A_{2g}{\rightarrow}^3T_{2g}(F)$ transition. Consequently, the electronic spectra of complexes **1**, **2**, and **3** are consistent with distorted $O_h$ geometry, the chromophore unit being of the $NiN_2O_4$ type.

Table 2. Electronic spectroscopic data for complexes **1-7**.

| No | Complex | Absorption band (kK) | Assigned transition |
|---|---|---|---|
| **1** | $[NiL(AcO)_2]$ | 8.90 ($\nu_1$)<br>11.90 ($\nu_2$)<br>15.20<br>18.20 ($\nu_3$) | $^3A_{2g}\rightarrow{}^3T_{2g}(F)$<br>$^3A_{2g}\rightarrow{}^3E_g(^3T_{1g})$<br>$^3A_{2g}\rightarrow{}^3B_{2g}(^3T_{1g})$<br>$^3A_{2g}\rightarrow{}^3T_{1g}(P)$ |
| **2** | $[Ni_2L(AcO)_4]$ | 9.09 ($\nu_1$)<br>12.14 ($\nu_2$)<br>14.63<br>17.95 ($\nu_3$) | $^3A_{2g}\rightarrow{}^3T_{2g}(F)$<br>$^3A_{2g}\rightarrow{}^3E_g(^3T_{1g})$<br>$^3A_{2g}\rightarrow{}^3B_{2g}(^3T_{1g})$<br>$^3A_{2g}\rightarrow{}^3T_{1g}(P)$ |
| **3** | $[Ni_3L_2(OH_2)_2(AcO)_4](AcO)_2$ | 8.21 ($\nu_1$)<br>11.48 ($\nu_2$)<br>15.68<br>18.15 ($\nu_3$)<br>24.96 | $^3A_{2g}\rightarrow{}^3T_{2g}(F)$<br>$^3A_{2g}\rightarrow{}^3E_g(^3T_{1g})$<br>$^3A_{2g}\rightarrow{}^3B_{2g}(^3T_{1g})$<br>$^3A_{2g}\rightarrow{}^3T_{1g}(P)$<br>charge transfer |
| **4** | $[CoLCl_2]$ | 6.15 ($\nu_2$)<br>15.35 ($\nu_3$) | $^4A_2\rightarrow{}^4T_1(F)$<br>$^4A_2\rightarrow{}^4T_1(P)$ |
| **5** | $[Co_2LCl_4]$ | 6.75 ($\nu_2$)<br>15.55 ($\nu_3$) | $^4A_2\rightarrow{}^4T_1(F)$<br>$^4A_2\rightarrow{}^4T_1(P)$ |
| **6** | $[Co_3L_2Cl_4]Cl_2$ | 6.70 ($\nu_2$)<br>15.40 ($\nu_3$) | $^4A_2\rightarrow{}^4T_1(F)$<br>$^4A_2\rightarrow{}^4T_1(P)$ |
| **7** | $[MnL(OH_2)_2Cl_2]$ | 14.89 ($\nu_1$)<br>17.24 ($\nu_2$)<br>19.44 ($\nu_3$) | $^6A_1\rightarrow{}^4T_{1g}$<br>$^6A_1\rightarrow{}^4T_{2g}$<br>$^6A_1\rightarrow{}^4A_{1g},{}^4E_g$ |

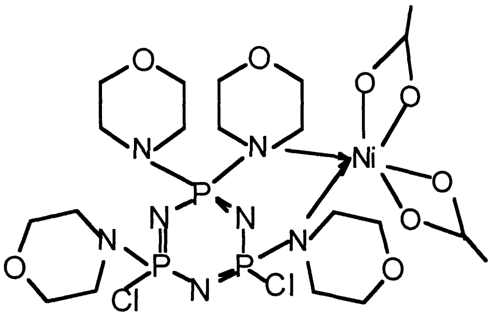

**1**

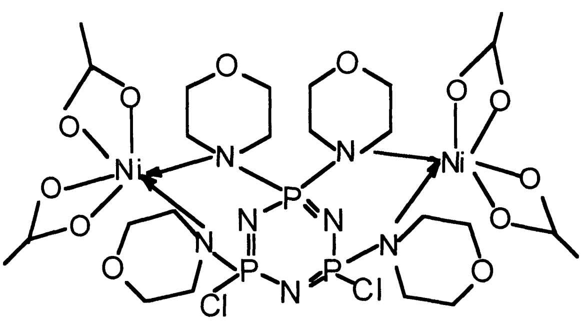

**2**

The complexes $[CoLCl_2]$ (**4**), $[Co_2LCl_4]$ (**5**) probably possess a distorted tetrahedral geometry of the metal ions, as confirmed by the presence of a large absorption band at 15.35 kK.[27] Generally, the tetrahedral Co(II) complexes show two large bands, $\nu_2$ and $\nu_3$, assigned to the spin allowed transitions $^4A_2{\rightarrow}^4T_1(F)$ and $^4A_2{\rightarrow}^4T_2$, respectively. The $\nu_3$ band is spin-forbidden and only rarely observed in tetrahedral Co(II) complexes.[27]

For the complex **7** and also, for the cobalt complexes reported here, the electronic spectra in the visible region appear quite similar to those of the corresponding chloro-complexes of the type $MCl_n^{x-}$.[28]

An octahedral geometry has also been assumed for compounds **6** and **7**, based on their electronic spectra (table 2).[27,28]

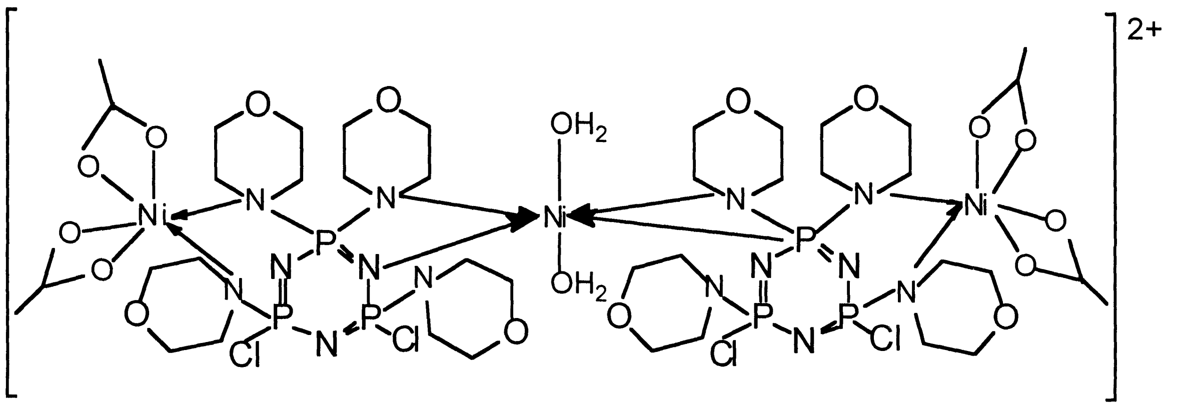

**3**

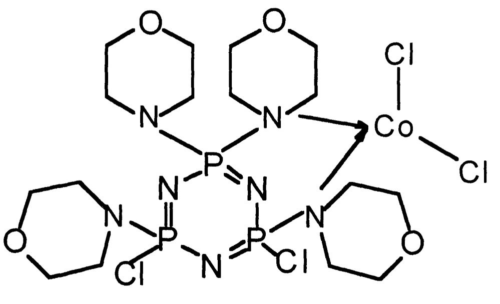

**4**

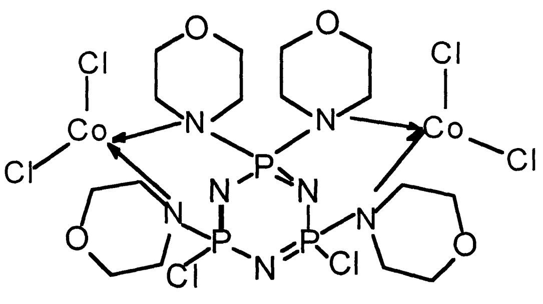

**5**

The IR spectra of the investigated compounds allowed us to confirm some assumptions regarding the co-ordination behaviour of the ligand. The following can be inferred from the obtained data:

〈 in the range specific for the $\nu_{P-N}$ vibration, the band appearing at 1230 $cm^{-1}$ in the free ligand was shifted to 1190 $cm^{-1}$ in all the prepared complexes. This has been interpreted as being due to the co-ordination of the metal ions through the exocyclic nitrogen atoms, since the co-ordination through phosphazenic (endocyclic) nitrogen(s) would lead to the splitting of the $\nu_{P-N}$ band [29,30];

〈 an influence of the metal ions has been observed on the $\rho_{CH2}$ band (appearing at 860 $cm^{-1}$ in the free ligand), which has been shifted to 840 $cm^{-1}$ in the prepared complexes which is probably due to the presence of the metal ions in the neighbourhood of the morpholine nitrogen atom, which in turn influenced the adjacent $CH_2$ moieties;

〈 the appearance of low intensity bands in the range 300-500 $cm^{-1}$, indicating the presence of M-X bonds;

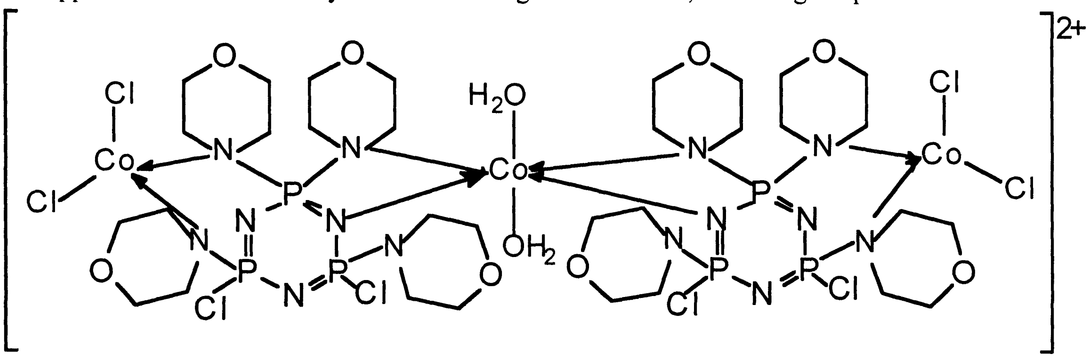

**6**

〈 for all the prepared complexes a band at 1620-1660 $cm^{-1}$ together with a very broad and intense one in the range of 3300-3500 $cm^{-1}$ were observed that are both absent in the free ligand. They were assigned to the $\delta_{HOH}$ stretching vibrations that prove the presence of co-ordination and lattice water molecules.

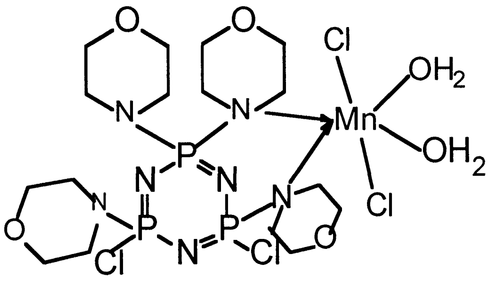

7

Table II: Antifungal activity of compounds **1-8** against several organisms.

| Compound | MIC (μg/mL) *A. flavus* C1150 | *A. niger* C418 | *Candida albicans* C316 |
|---|---|---|---|
| **1** | 25 | 29 | 16 |
| **2** | 28 | 34 | 17 |
| **3** | 32 | 39 | 16 |
| **4** | 29 | 30 | 18 |
| **5** | 33 | 35 | 19 |
| **6** | 35 | 37 | 20 |
| **7** | 9 | 10 | 2 |
| **Ketoconazole 8** | 1.2 | 1.8 | 0.06 |

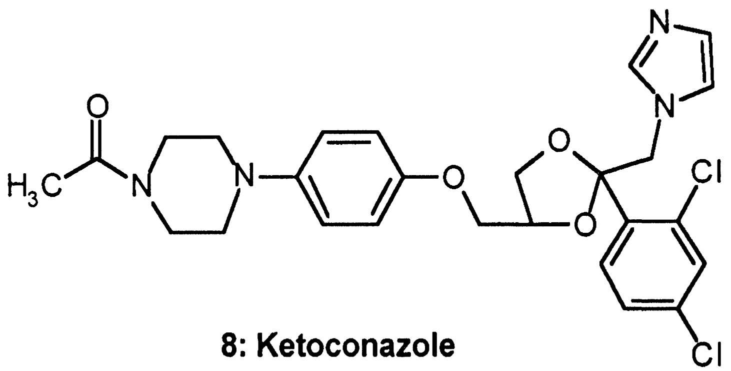

**8: Ketoconazole**

Biological activity data of the new derivatives **1-7** as compared to the standard antifungal ketoconazole **8** are shown in Table 3.

From the data of Table 3, one should note that the new compounds **1-7** reported here represent a new class of antifungals, with a lower biological activity as compared to that of ketoconazole against the investigated organisms, but with MIC-s in the micromolar range, which might induce strong antifungal effects.

The most active derivative was the Mn(II) complex **7**, followed by the Ni(II) and the Co(II) complexes (the less active). Generally the mononuclear complexes were more active than the dinuclear ones, which in turn were more active than the trinuclear ones. *Candida* was most susceptible to inhibition, followed by *A. flavus*, whereas *A. niger* was the most resistant to this type of antifungals. In this respect, the complex derivatives parallel the biological activity of ketoconazole, although they are much less active.

Ketoconazole **8** is known to act as an inhibitor of lanosterol 14-α-demethylase (CYP51A1), a microsomal cytochrome P-450 dependent enzyme system belonging to a gene superfamily involved in sterol biosynthesis in fungi, plants and animals.[18-21] CYP51A1 has been shown to catalyse the conversion of lanosterol to the 14-desmethylated derivative, ergosterol, through a complicated oxidative sequence. Its inhibition in fungi causes the depletion of ergosterol and accumulation of 14-methylsterols in the membrane of the cells, disturbing thus membrane function and causing the death of these organisms.[18,19]

Table 4: Levels of ergosterol in *C. albicans* cultures after treatment with different concentrations of the azole CYP51A1 inhibitor ketoconazole and complex 7.

| Inhibitor | Concentration (μg/mL) | % Ergosterol* |
|---|---|---|
| Ketoconazole | 0.01 | 89 ± 5 |
| Ketoconazole | 0.02 | 66 ± 7 |
| Ketoconazole | 0.05 | 8 ± 4 |
| 7 | 0.25 | 96 ± 3 |
| 7 | 0.50 | 70 ± 5 |
| 7 | 1.00 | 28 ± 6 |
| 7 | 1.50 | 7 ± 2 |

*Mean ± standard deviation (n = 3); The amount of ergosterol present in the same amount of wet cells from the culture grown in the absence of inhibitor is taken as 100 %.

In order to test the hypothesis that the compounds reported here act as ergosterol biosynthesis inhibitors, similarly to the azole antifungals, the amounts of ergosterol present in *C. albicans* cultures after treatment with different concentrations of new the inhibitor **7** and ketoconazole **8**, a potent CYP51A1 inhibitor,[19] have been determined by means of a HPLC method (Table 4).[22]

These data show that at low concentrations of inhibitor, around 66-96 % of ergosterol (as compared to the amount of sterol formed in cultures in which inhibitors have not been added, and which was considered 100%) is still synthesised. By increasing the concentrations of inhibitors used in the experiments, the amount of synthesised ergosterol decreased dose-dependently. A similar effect has been observed for the well-known CYP51A1 inhibitor Ketoconazole **8** as well as for the complex **7** synthesised in the present study. These data allow us to propose a similar mechanism of action for the two classes of antifungal compounds, i.e., the inhibition of lanosterol-14-α-demethylase, although it is not improbable that our compounds might interfere with other enzyme(s) involved in the ergosterol biosynthetic pathway.

**References**

1. H.R. Allcock, S. Al-Shali, D.C. Ngo, K.B. Visscher, M. Parvez, *J. Chem. Soc. Dalton Trans.*, **1995**, *21*, 3521-3532.
2. H.R. Allcock, S.E. Kuharcik, K.B. Visscher, D.C. Ngo, *J. Chem. Soc. Dalton Trans.*, **1995**, *17*, 2785-2796.
3. H.R. Allcock, *Phosphorus, Sulfur Silicon Relat. Elem.*, **1993**, *76*, 199-202.
4. H.R. Allcock, D.C. Ngo, M. Parvez, K.B. Visscher, *J. Chem. Soc. Dalton Trans.*, **1992**, *10*, 1687-1700.
5. H.R. Allcock, *Phosphorus, Sulfur Silicon Relat. Elem.*, **1989**, *41*, 119-134
6. K.R.J. Thomas, V. Chandrasekar, S. Scott, R. Hallford, W. Cordes, *J. Chem. Soc. Dalton Trans.*, **1993**, *17*, 2589-2594
7. T.G. Meyer, P.G. Jones, R. Schmutzler, *Z. Naturforsch. B*, **1992**, *47*, 517-525.
8. P. Wisian-Neilson, M. Islam, M.A. Schaefer, *Phosphorus, Sulfur Silicon Relat. Elem.*, **1989**, *41*, 135-140.
9. J.E. Mark, H. R. Allcock, R.West, "Inorganic Polymers", Prentice & Hall Inc., New Jersey, **1992.**
10. G.A. Carriedo, L. Fernandez-Catuxo, F.G.J. Alonso, P. Gomez-Elipe, *J. Organomet. Chem.*, **1995**, *503*, 59-68
11. R. Hasselbring, H.W. Roesky, A. Heine, D. Stalke, G.M. Sheldrick, *Z. Naturforsch. B*, **1994**, *49*, 43-49
12. A. Chandrasekaran, S.S. Krishnamurthy, M. Nethaji, *J. Chem. Soc. Dalton Trans.*, **1994**, *1*, 63-68.
13. P. Wisian-Neilson, K.T. Nguyen, T. Wang, S. Rippstein, C. Claypool, F. J. Garcia-Alonso, *Phosphorus, Sulfur Silicon Relat. Elem.*, **1994**, *87*, 277-286.
14. M. Veith, M. Kross, J.F. Labarre, *J. Mol. Struct.*, **1991**, *243*, 189-209.
15. C. Guran, M. Barboiu, A. Meghea, I. Jitaru, M. Cimpoesu, D. Berger, P. Diaconescu, M. Bojin, V. Iluc, I. Bita, *Rev. Roum. Chim.,* **1997**, *43,* 7-11.
16. M. Barboiu, C. Guran, I. Jitaru, M. Cimpoesu, C. T. Supuran, *Metal Based Drugs*, **1996**, *3*, 233-240.
17. A. Boye, Ph.D. Thesis, *Synthesis and characterisation of nanofiltration polyphosphazene membranes*, **1992**, Université de Montpellier, France

18. a) D. Sanglard, F. Ischer, L. Koymans, J. Bille, *Antimicrob. Agents Chemother.*, **1998**, *42*, 241-253; b) C.A. Hitchcock, S.B. Brown, E.G. Evans, D.J. Adams, *Biochem. J.*, **1989**, *260*, 549-556; c) C.T. Supuran, A. Scozzafava, F. Briganti, G. Loloiu, O. Maior, *Eur. J. Med. Chem.*, **1998**, *33*, in press; d) C.T. Supuran, A. Scozzafava, F. Briganti, G. Loloiu, O. Maior, *J. Enzyme Inhib.*, **1998**, *13*, in press ; e) F. Briganti, A. Scozzafava, C.T. Supuran *Eur. J. Med. Chem.*, **1997**, *32*, 901-910.
19.a) H. Vanden Bossche, *Drug Dev. Res.*, **1986**, *8*, 287-298; b) P. Marichal, H. Vanden Bossche, *Acta Biochim. Pol.*, **1995**, *42*, 509-516; c) F.C. Odds, *J Antimicrob. Chemother.*, **1993**, *31*, 463-471.
20. M. Barboiu, M. Cimpoesu, C. Guran, C.T. Supuran, *Metal Based Drugs,* **1996**, *3*, 227-232.
21. O.S. Kinsman, D.G. Livermore, C. Smith, *Antimicrob. Agents Chemother.*, **1993**, *37*, 1242-1246.
22. a) E. Hansbury, T.J. Scallen, *J. Lipid Res.*, **1978**, *19*, 742-746; b) B. Ruan, N. Gerst, G.T. Emmons, J. Shey, G.J. Schroepfer, *J. Lipid Res.*, **1997**, *38*, 2615-2626.
23. a) Y. Aoyama, Y. Yoshida, Y. Sonoda, Y. Sato, *Biochim. Biophys. Acta,* **1991**, *1081*, 262-266; b) Y. Aoyama, Y. Yoshida, Y. Sonoda, Y. Sato, *Biochim. Biophys. Acta,* **1992**, *1122*, 251-255.
24. a) Y. Yoshida, Y. Sonoda, *Biochem. Biophys. Res. Commun.*, **1992**, *183*, 1266-1272; b) J.M. Trzaskos, S.S. Ko, R.L. Magolda, M.F. Favata, R.T. Fischer, S.H. Stam, P.R. Johnson, J.L. Gaylor, *Biochemistry* **1995**, *34*, 9670-9676.
25. Hyperchem for Windows, available from Serena Software, Box 3076, Bloomington, IN 47402-3076, USA.
39. L. Sacconi, F. Mani, A. Bencini, "Nickel", in "Comprehensive Coordination Chemistry", G. Wilkinson, R. Gillard, J. McCleverty Eds., Pergamon Press, Oxford, **1987**, Vol. 5, pp. 1-347.
40. L. Banci, A. Bencini, C. Benelli, D. Gatteschi, C. Zanchini, *Struct. Bonding*, **1982**, *52*, 37-79.
28. A.B.P. Lever, "Inorganic Electronic Spectroscopy", Elsevier Publ. Comp., Amsterdam, London, New York, **1984.**
29. R. Nakamoto, "Infrared and Raman Spectra of Inorganic and Coordination Compounds", Willey, New York, **1986**
30. K.R.J. Thomas, P. Tharmaraj, V. Chandrasekhar, C.D. Bryan, A.W. Cordes, *Inorg.Chem.*, **1994**, *33*, 5382.

**Received: June 29, 1998 - Accepted: July 30, 1998 - Received in revised camera-ready format: October 2, 1998**

